# Public willingness to receive chlamydia, gonorrhea, syphilis, and trichomoniasis vaccines: a scoping review

**DOI:** 10.1186/s12913-023-10334-9

**Published:** 2023-11-23

**Authors:** T. Valleroy, Colin Garon, Janamarie Perroud, Abram L. Wagner

**Affiliations:** 1 Department of Epidemiology, University of Michigan, Ann Arbor, MI 48109 USA; 2 Department of Anthropology, University of Michigan, Ann Arbor, MI 48109 USA; 3 Department of Health Management and Policy, University of Michigan, Ann Arbor, MI 48109 USA

**Keywords:** Chlamydia trachomatis, Neisseria gonorrhoeae, Treponema pallidum, Sexually Transmitted Diseases, Vaccine acceptability

## Abstract

**Background:**

Sexually transmitted infections (STIs) like chlamydia, gonorrhea, syphilis, and trichomoniasis contribute significantly to global morbidity and mortality. Researchers are pursuing vaccines for these STIs, and a clinical trial is currently underway for a chlamydia vaccine. However, there is little research available on individuals’ willingness to receive chlamydia, gonorrhea, syphilis, and trichomoniasis vaccines. The purpose of this analysis was to map the existing literature we have on individuals’ willingness to receive these bacterial/parasitic STI vaccines and understand what information on vaccine acceptability is still needed.

**Methods:**

We searched seven databases for literature on STI vaccine acceptability, then conducted title/abstract and full-text reviews to assess eligibility. All reviews and abstractions were conducted blindly by two reviewers, with discrepancies settled by discussion or the input of a third reviewer.

**Results:**

Eight of the original 2,259 texts of interest met inclusion criteria. After data abstraction, we found that gonorrhea was the most commonly examined, followed by chlamydia and syphilis. Trichomoniasis vaccine acceptability was not reported. Most texts reported high acceptability, but there did not appear to be data describing how vaccine characteristics affect acceptability. Similarly, while the literature covers a variety of populations, most of the study populations were based out of the United States or Canada and were patrons of healthcare facilities or participants from a larger health intervention study. Therefore, more information is needed on populations outside North America, and on groups with lower healthcare access and utilization.

**Conclusion:**

As the incidence of bacterial and parasitic STIs increase, and as we grow nearer vaccines for these illnesses, understanding how likely the public is to accept and receive these vaccines is crucial to their success. While the existing literature describes STI vaccine acceptability in a variety of populations, their overall number is small. More research into STI vaccine acceptability outside of North America, and especially examining how factors like number of doses, timing, and cost influence vaccine acceptability is needed to ensure effective future vaccine rollouts.

**Supplementary Information:**

The online version contains supplementary material available at 10.1186/s12913-023-10334-9.

## Introduction

Sexually Transmitted Infections (STIs) are a persistent and prevalent threat to health globally. Chlamydia, gonorrhea, syphilis, and trichomoniasis account for a significant proportion of STI cases. In 2016 alone, there were an estimated 127.2 million new chlamydia cases, 86.9 million new gonorrhea cases, 6.3 million new syphilis cases, and 156.0 million new trichomoniasis cases worldwide [1]. Together, these four diseases averaged 1 million new infections every day [1].

These infections can cause serious disease. Their effects range from genital ulceration and Pelvic Inflammatory Disease (PID) to cardiovascular and neurological disease [1]. Pregnant people with chlamydia and gonorrhea have a higher risk of ectopic pregnancy and infertility [1], trichomoniasis is associated with increased risk of preterm delivery and prelabour rupture of membranes [2] and syphilis infections can lead to reduced fetal growth, spontaneous abortions, and perinatal deaths [3]. This is particularly concerning, given the high rates of STIs among young, reproductive-age people. In the United States, for example, adolescents aged 15–24 years old, make up a quarter of the population, but account for half of new STI cases annually [4].

Although chlamydia, gonorrhea, syphilis, and trichomoniasis are all “curable” with a course of antibiotics, they still spread readily in the population and contribute to significant health impacts for millions every year. Many cases of these STIs are asymptomatic or mildly symptomatic [5], leading to underdiagnosis and treatment. Social stigma around sexual practices and disparities in access to testing and treatment further exacerbate this problem [6–8].

Antimicrobial resistance (AMR) is also a rising problem for supposedly curable STIs, gonorrhea especially [9]. Gonorrhea strains have already developed resistance to sulphonamides, penicillins, tetracyclines, macrolides, fluoroquinolones, and early-generation cephalosporins [9],. In the United States alone, half of gonorrhea cases in 2018 were ARM resistant [10]. This rapid escalation has made our ability to treat the 86.9 million new gonorrhea infections every year increasingly tenuous.

Interventions like screening, sexual education, and condom advocacy [6] have had some success at increasing STI diagnosis and reducing spread, they have not yet been enough to mitigate the rampant incidence of STIs. Vaccines could be an additional tool for control of STIs. Already, the human papillomavirus (HPV) and hepatitis B vaccines – which protect against STIs – have had success in reduce associated morbidity and mortality. Other STI vaccines are on the horizon. In 2019 Abraham et al. published the results for the Phase I Trial of their chlamydia vaccine candidate [11], and in recent years, there have been several trials examining the efficacy of *Neisseria meningitidis* vaccine at preventing *Neisseria gonorrhoeae* [12, 13]. Mathematical models predicting the epidemiological impact of gonorrhea, chlamydia, and syphilis vaccines support this vaccine development push, indicating that effective vaccines could significantly reduce disease prevalence beyond existing interventions [14–17].

However, the existence of a vaccine alone does not ensure coverage–attitudes towards STIs and vaccines are likely to influence STI vaccine uptake. Parents may have substantial dispreferences for STI vaccines versus non-STI vaccines [18]. Additionally, parental concerns about vaccine safety and appropriateness, individuals’ sense of susceptibility, societal stigma around sexual activity, media misinformation, lack of awareness about vaccination, and degree of provider attitudes and vaccine endorsement are all frequently cited factors that contribute to hesitancy around both existing and hypothesized STI vaccines [19–21].

Vaccine acceptability research is therefore crucial to anticipating public hesitancy for future STI vaccines. This need for research on how to roll-out an STI vaccine is already reflected in reports from the WHO [22] and other researchers [23]. With a chlamydia vaccine already in clinical trials and the rising evidence that meningococcal vaccines provide partial protection against gonorrhea [12, 13, 24], there is a need to understand the scope of what we already know about STI vaccine acceptance.

The purpose of this scoping review is to identify existing studies examining chlamydia, gonorrhea, syphilis, and trichomoniasis acceptability, map out their content, and identify populations and contexts that remain unstudied. By identifying what evidence we have and what gaps exist, we hope to provide direction for future research and for the effective implementation of STI vaccine programs.

## Methods

We modeled the protocol for this scoping analysis after Arksey and O’Malley’s framework [25], guided also by Levac et al.’s recommended enhancements [26] and the scoping review practices outlined in JBI’s Manual of Evidence Synthesis [27]. The protocol for this scoping analysis is publicly available [28].

### Inclusion and exclusion criteria

We established a set of inclusion and exclusion criteria centered around the characteristics of a texts’ participants, concepts, and contexts to evaluate if texts returned by our searches were relevant to this review. Texts examining human participants’ willingness to receive chlamydia, gonorrhea, syphilis, and trichomoniasis vaccines for themselves, or their willingness to have their children/dependents vaccinated, were eligible for inclusion. To be eligible, texts also had to assess participants’ willingness to receive, or have one of their children/dependents receive, one or more of the vaccines of interest. Studies examining attitudes towards the disease itself or non-vaccination interventions were ineligible. Texts discussing researchers’ interest in developing or implementing the vaccines were excluded, as were texts examining healthcare worker’s willingness to recommend or provide vaccines.

To gather as many relevant texts as possible, studies examining human populations in any context were eligible for inclusion–opinions towards vaccines for bacterial/parasitic STIs in any geographic regions, cultures, communities, and focus groups were of interest. Any text published before database searches were initiated on August 8, 2022, were eligible for inclusion.

Table 1 documents the complete inclusion criteria for the scoping review.

**Table 1:** Inclusion and exclusion criteria for the review

	Inclusion	Exclusion
Participants	• Study population is composed of any human participants	
Vaccines of interest	• Includes chlamydia, gonorrhea, syphilis, and/or trichomoniasis vaccines	• Does not include disaggregated measures of interest for chlamydia, gonorrhea, syphilis, and/or trichomoniasis vaccines
Concept	• Discusses participants’ willingness to receive one or several of the vaccines of interest • Discusses participants’ willingness to have a child/ward vaccinated with one or several of the vaccines of interest	• Does not specify which STIs are being examined
Context	• Studies from any geographic context and any time before August 9th, 2022	• Any studies published after August 9th 2022

### Types of study

For this synthesis, we considered studies of any research design and publication type for inclusion, including both qualitative and quantitative studies. Applicable evidence syntheses, like systematic and scoping reviews, along with conference abstracts and non-peer-reviewed literature were also eligible for inclusion.

### Identifying relevant studies

We used a three-step search strategy [19] to test and refine our initial search terms in Scopus® and Pubmed®, conduct our final searches to identify texts of interest, and perform a reference search on our included texts to identify relevant texts we might have missed during the initial searches. We conducted our searches in PubMed®, Embase®, Scopus®, Cochrane Library®, PsychInfo®, Global Index Medicus, and Google Scholar®; all searches were conducted between August 8, 2022, and August 9, 2022. Most search terms were limited to title and abstract or title, abstract, and keyword searches, though given time limitations, the Google Scholar® search was limited to titles only. A copy of the Scopus® search strategy is available in Additional File 1. We exported all of the identified texts from our searches to Zotero (Corporation for Digital Scholarship, Vienna, Virginia), where we combined them into a singular list and checked them for duplicates before exporting them to Rayyan (Rayyan Systems Inc., Cambridge, MA), where we again checked for duplicates.

### Study selection

Once we completed deduplication, reviewers C.G. and T.V. conducted a blinded pilot of the eligibility criteria on the abstracts of 25 randomly selected texts and achieved the > 75% agreement necessary to proceed with abstract reviews for all identified texts.

The reviewers conducted independent, blinded title and abstract reviews in Rayyan. Once abstract reviews were completed, the reviewers unblinded and resolved any disagreements through discussion or the opinion of a third reviewer, A.W. The reviewers acquired full text copies of any articles that were marked as “include” or “maybe” after discussion and conducted full text reviews to determine inclusion or exclusion from the synthesis. All disagreements were settled through discussion or the opinion of the third reviewer.

### Charting the data

We developed a data abstraction form, which included title, year of publication, country of origin, country where the study was conducted, aims/purpose, population of interest, sample size and description, methodology, intervention, outcomes, and key findings. We expanded the extraction template to include a section for collecting information on the questions used to examine vaccine acceptability, including question(s) used, response scale used, if the acceptability measure was based on one question or an aggregate, and the word or phrase used to describe acceptability. Reviewers T.V. and C.G. conducted concurrent abstractions for each included text.

We also used a critical appraisal tool to examine the overall quality of the included studies. Given that the results of interest in the included studies were descriptive statistics for STI vaccine acceptability, we limited our evaluation to questions 1, 2, 7, and 8 of the JBI Critical Appraisal Tool for Analytical Cross Sectional Studies [27], with additional clarifying criteria established by the review team (Additional File 2).

### Collating, summarizing and reporting the results

We used the abstraction data to map the included studies’ characteristics and designs, as well as the relevant findings about vaccine acceptability. We then conducted basic thematic analyses of the survey instruments and questionnaires to provide insight into how investigators inquired about willingness to receive chlamydia, gonorrhea, syphilis, and/or trichomoniasis vaccines. For texts where the survey materials and/or vaccine acceptability questions were not available in the text or additional files, or where the geographic context of the research was unclear, investigators reached out to the texts’ authors to inquire about the questions utilized and geographic context. As of November 6, 2022, we had not received clarifying information on survey questions or study locations.

## Results

### Excluded texts

**Fig. 1:**
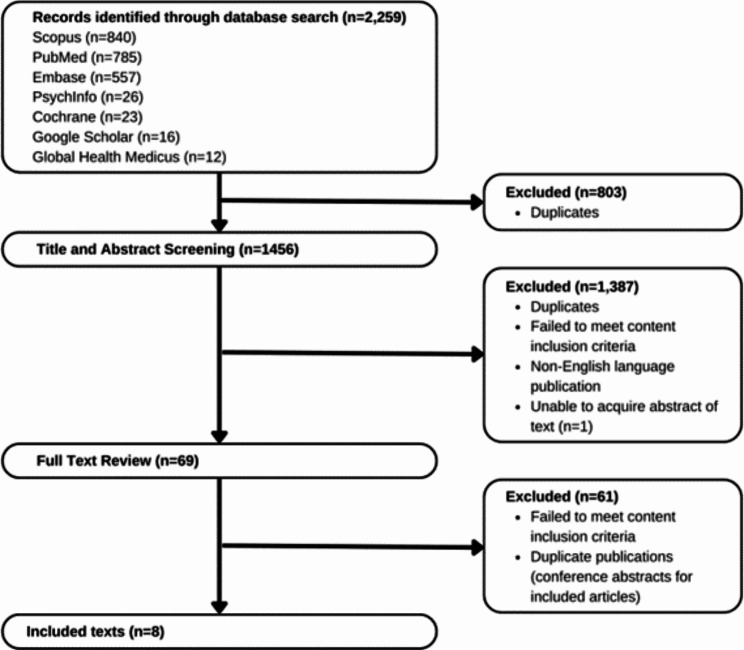
Diagram for retrieved, screened, reviewed, and included texts with counts for texts examined or excluded at each step

We collected 2,259 texts of interest from our database searches. 803 were excluded because they were duplicate copies, and another 1,387 were excluded after the title and abstract review process. Of the 69 texts that went through full-text reviews, eight met inclusion criteria (Fig. 1).

Most texts were excluded because they did not meet inclusion criteria, usually because they did not examine at least one of the diseases of interest, did not examine vaccine acceptability, or did not examine the vaccines of interest in human populations. One-hundred additional texts were excluded because they were not published in English. Two texts were excluded because they presented the same results as other included texts in the form of pre-publication conference abstracts [29, 30]; in both instances, the abstracts were excluded while the full articles were included. Two more texts were excluded because they did not specify an STI but instead referred more abstractly to overall STI vaccine acceptability [31, 32]. One other paper was excluded because it assessed the perceived “importance” of a vaccine rather than participants’ “willingness” to receive the vaccines [33].

**Table 2:** Description of studies included in the scoping review

Publication	Country	Study Setting/Frame	Population of Interest	STI of Interest	Sample Size	Results of Interest
Abara 2022 [34]	United States	Participant pool for the 2019 American Men’s Internet Survey (AMIS)	Men who have sex with men (MSM)	Gonorrhea	n = 4951	**Willingness to accept a Gonorrhea vaccine** Very willing: 63.6% Somewhat willing: 19.9% Neither willing nor unwilling: 7.5% Did not know: 5.1% Somewhat unwilling: 1.5% Very unwilling: 2.4%
Trent 2016 [35]	United States*	Participant pool for a Pelvic Inflammatory Disease Clinical trial	Adolescents and young adults (ages 13–25)	Chlamydia	n = 106	**% Willingness to receive a Chlamydia vaccine** 93%
Zimet 2002 [36]	United States*	A Pediatric Primary Care Setting	Parents and their adolescent children (ages 12–17)	Gonorrhea	**Parents** n = 50 **Adolescents** n = 50	**Parents’ acceptability of a Gonorrhea vaccine for their child** > 4 on a 5 point scale **Adolescents’ acceptability of a Gonorrhea vaccine** > 4 on a five point scale
Bonney 2007 [37]	United States	The Rhode Island Adult Correctional Institute	Incarcerated women	Gonorrhea	n = 106	**% Willing to accept a Gonorrhea Vaccine** 79%
de Waal 2022 [38]	Canada	Participant Pool for the Quadrivalent HPV Vaccine Evaluation Study (QUEST) Cohort	HPV-vaccinated women	Gonorrhea Chlamydia Syphilis	n = 1092	**% Interested or very interested in receiving a vaccine for** Syphilis: 78.2% (95% CI 75.6–80.6) Chlamydia: 80.3% (95% CI 77.8–82.6) Gonorrhea: 78.3% (95% CI 75.7–80.7)
Plotnikoff 2020 [39]	Canada	Two STI clinics in Vancouver, Canada	STI clinic users	Gonorrhea Chlamydia Syphilis	n = 293	**% Interested or very interested in receiving a vaccine for** Syphilis: 76% Chlamydia: 74% Gonorrhea: 76%
Mays 2004 [40]	United States	A suburban private practice and an urban hospital clinic in Marion County, Indiana	Parents of children (ages 8–17)	Gonorrhea	n = 34	**% Accepting of a Gonorrhea vaccine for their child** 76%
Zimet 2005 [41]	United States*	Urban adolescent health clinics and private pediatric practices	Parents and their adolescent children (ages 12–17)	Gonorrhea	**Parents** n = 320 **Adolescents** n = 320	**% Parents who agreed or strongly agreed they would get their child vaccinated against Gonorrhea** 85.3% **% Adolescents who agreed or strongly agreed they would get vaccinated against Gonorrhea** 89.0%

*Texts that did not directly state the geographical context of their study, but whose authors had affiliations within the United States

### Description of study populations

The majority of included studies were conducted and published in either the United States or Canada, though the country of investigation was unclear in three studies (Table 2). The authors of each of those three studies were affiliated with institutions in the United States (Table 2). The texts’ survey populations included parents of children or parent-adolescent pairs [36, 40, 41], men who have sex with men (MSM) [34], adolescents and young adults from a Pelvic Inflammatory Disease (PID) trial [35], incarcerated women [37], HPV-vaccinated women [38], and STI clinic users [39]. The participants for all of the included studies were either sampled from healthcare settings, participant pools from larger health-related studies, or both (Table 2). For example, Bonny et al.’s 2007 study on incarcerated women’s willingness to receive gonorrhea vaccines was nested in a alcohol use and Human Immunodeficiency Virus (HIV) risk behavior reduction trial [37].

### Critical appraisals

We conducted critical appraisals concurrently with abstraction. Criteria included whether or not inclusion and exclusion criteria for participants were clearly defined; whether or not the study setting and participants were well described, including study time and location; whether or not it was clear how vaccine acceptability was measured and whether or not information on the survey tools’ sources or development were provided; and whether or not appropriate statistical analysis were used, including a probability measure of a Type I error (e.g., p-value or confidence interval) [Additional File 2] [27]. While most texts were missing at least one assessment component, no text had to be excluded because it did not meet any of our assessment criteria [Additional File 2]. Texts most often omitted an explanation of what survey tool they used to assess vaccine acceptability or how they developed their acceptability question(s)–thus not speaking to their tools’ validity–or they did not provide a precision or dispersion measure for their vaccine acceptability estimates [Additional File 2].

### Text characteristics

Each of the eight included texts describe cross-sectional studies [34–41]. Six are journal articles [34, 37–41] while two are abstracts [35, 36], and all of the studies were published since 2000 (Table 2). Interestingly, the papers seem to be clustered at different time intervals, with half published between 2002 and 2007 and half published between 2016 and 2022. This is likely due to the fact that several of the papers have overlap in authorship or come from related studies. Zimet et al. (2002)’s population [36], specifically, is a subset of Zimet et al. (2005)’s population [41], which includes both Zimet et al. (2002)’s participants as well as additional participants recruited afterwards. After deliberation, the review team decided that because their results were distinct, both were eligible for inclusion.

### Diseases of interest and vaccine acceptability

The majority of included studies examined gonorrhea vaccine acceptability, three examined chlamydia, and two examined syphilis (Table 2). None of the included texts presented trichomoniasis vaccine acceptability in their results, though both de Waal et al. (2022) and Plotnikoff et al. (2020)’s supplemental materials showed questions about trichomoniasis in their survey materials [38, 39].

The presentation of results varies between studies, with some reporting percentages of the participants who would accept a vaccine or not, the percentages of the participants who fall in different willingness categories, the average vaccine acceptability scores across participants, or a combination of these indicators (Table 2). All of the studies indicated relatively high acceptability for their respective vaccines, though most do not present variance or confidence estimates (Additional File 2).

Abara et al. (2022) found that 63.6% of MSM respondents were very willing to accept a gonorrhea vaccine, 19.9% were somewhat willing, 7.5% were neither willing nor unwilling, 5.1% did not know, 1.5% were somewhat unwilling, and 2.4% were very unwilling (Table 2) [34]. Zimet et al. (2002) reported that both parents’ acceptability of a gonorrhea vaccine for their child and adolescents’ acceptability of a gonorrhea vaccine was, on average, > 4 points on a five point scale where higher scores indicate higher acceptability [36]. Zimet et al. (2005)’s later publication on an expanded participant pool found that 85.3% of parents agreed or strongly agreed they would get their child vaccinated against gonorrhea and 89.0% of adolescents agreed or strongly agreed they would get vaccinated against gonorrhea [41]. Meanwhile, Mays et al. (2004) found that a slightly more modest 76% of parents would accept a gonorrhea vaccine for their child [40]. Bonney et al. (2007) found that among their sample of incarcerated women, 79% were willing to accept a gonorrhea vaccine [37], while 78.3% (95% CI 75.7–80.7) of de Waal’s sample of HPV-vaccinated women [38] and 76% of Plotnikoff et al. (2020)’s STI clinic-users indicated interest in a gonorrhea vaccine [39].

Only Trent et al. (2016), de Waal et al. (2022), and Plotnikoff et al. (2020) reported chlamydia vaccine acceptability. Trent et al. (2016) found that 93% of their adolescent and young adult sample were willing to receive a chlamydia vaccine [35], while 80.3% (95% CI 77.8–82.6) of de Waal et al. (2022)’s sample [38] and 74% of Plotnikoff et al. (2020)’s sample [39] were interested in receiving a chlamydia vaccine (Table 2).

Plotnikoff et al. (2020) and de Waal et al. (2022), which have considerable overlap in authorship and the latter of which adapted the survey used in the former, were the only two studies to examine syphilis vaccine acceptability [38, 39]. Plotnikoff et al. (2020) found that 76% of participants were interested or very interested in receiving a vaccine for syphilis, while 78.2% (95% CI 75.6–80.6) of de Waal et al. (2022)’s participants were interested or very interested in receiving the vaccine.

**Table 3:** Information on the survey methods and specific questions used for each study

First Author and Publication Year	Survey Delivery Method	Question Used	Scale
Abara 2022 [34]	Self-administered Questionnaire (paper)	“If a gonorrhea vaccine is available, how willing would you be to get a vaccine that would protect you against gonorrhea?“	A six-point scale with responses “do not know”, “very willing”, “somewhat willing”, “neither willing nor unwilling”, “somewhat unwilling” and “very unwilling”.
Trent 2016 [35]	Interview	*	*
Zimet 2002 [36]	Self-administered Questionnaire	*	A five-point scale, with 5 indicating the highest acceptability
Bonney 2007 [37]	Interview	“Please tell us which number best describes your willingness to receive a vaccine for gonorrhea if a safe and effective vaccine for gonorrhea was available”	A 12-point scale where 0 indicated “I would never get this vaccine and 11 indicated “I would definitely get this vaccine”. Data was later dichotomized so scores 8–11 indicated a “yes” response.
de Waal 2022 [38]	Self-administered questionnaire (digital)	“If available today, I would be interested in receiving a vaccine to prevent the following STI’s”	A five-point scale with responses “not at all interested”, “not very interested”, “neutral”, “interested”, and “very interested”.
Plotnikoff 2020 [39]	Self-administered questionnaire (paper)	“If available today, I would be interested in receiving a vaccine to prevent STIs.”	A five-point scale with responses “very uninterested”, “not interested”, “neutral”, “interested”, and “very interested”.
Mays 2004 [40]	Interview	*	*
Zimet 2005 [41]	Self-administered interview	*	A five-point scale with responses ranging from “strongly disagree” to “strongly agree”

*Information unavailable

As shown in Table 3, four of the included studies used either paper or digital questionnaires to assess vaccine acceptability, and four used interviews, one of which was recorded and self-administered [41].

For the studies whose questions were available, most used the terms “willing”, “willingness”, or “interest” to characterize participants’ attitudes towards receiving a vaccine(s) (Table 3). Scales ranged from five-point to twelve-point Likert scales that represented participant willingness, acceptance, interest, or agreement, and several were dichotomized during analysis to create a binary variable that represented willingness and unwillingness to receive vaccines.

### Factors associated with vaccine acceptability

Several studies also assessed factors associated with willingness and reasons for acceptance. All of the studies examining parental opinions found that child age did not significantly impact parental willingness to have their children vaccinated against gonorrhea [36, 40, 41]. However, while Zimet et al. (2002) and Zimet et al. (2005) found that parental education did not have a significant impact on vaccine acceptability [36, 41], Mays et al. (2004) found that parents with lower education were more likely to be accepting [40]. Both Zimet et al. (2005) and Mays et al. (2004) agreed that parents utilizing public health clinics were significantly more likely to have their children vaccinated than those using private practices [40, 41].

Abara et al. (2022) reported that among MSM, younger men and men with a high school diploma/GED or higher education were significantly more likely to receive a gonorrhea vaccine, but no significant differences emerged by race [34]. Abara et al. (2022) also found that men who reported condomless anal sex (CAS), preexposure prophylaxis (PrEP) use, having HIV, testing for a bacterial STD, or having a bacterial STI in the past 12 months were more likely to receive a gonorrhea vaccine. Trent (2016) similarly found that adolescents with a history of chlamydia were more likely to receive a chlamydia vaccine [35].

Bonney et al. (2007) found that in their sample of incarcerated women, demographic characteristics like age, race, and education were not correlated with vaccine acceptability, but psychosocial vaccine correlates like a greater perceived severity of gonorrhea, a greater vulnerability to gonorrhea, and lower vaccine fear were positively associated with vaccine acceptance [37]. Both Plotnikoff et al. (2020) and de Waal et al. (2022) found that participants’ desire to protect themselves and their partners were the first a second most important factors driving acceptability [29, 30], while Mays et al. (2004) similarly found that a desire to protect one’s children and others and concern about disease characteristics were frequently cited by accepting parents [31]. Plotnikoff et al. (2020) and de Waal et al. (2022) both also found potential vaccination cost to be the most frequently identified barrier to vaccination [38, 39].

When examining topics around vaccine guidance and receipt, Trent et al. (2016) reported that health care providers were the most significant sources of vaccine recommendation for participants, ahead of parents, friends, and partners [35]. Abara et al. (2022) similarly reported that participants preferred locations to receive a gonorrhea vaccination would be their primary care provider’s office or an STI clinic [34], which is supported by Plotnikoff et al. (2020), who reported that STI clinics were their participants’ preferred place to receive a vaccine [39].

## Discussion

Future roll-outs of STI vaccines could be an important tool in addressing the substantial burden of these diseases. This scoping review details the range of studies on acceptance of future vaccines for bacterial and parasitic diseases. Overall, the eight studies included in this analysis indicate relatively high levels of STI vaccine acceptability in the populations studied. A desire to protect oneself, one’s child, and one’s partner were common acceptability motivators [38–40], and concerns about STIs and histories of STI infection were related to vaccine acceptability in several studies [34, 35, 37, 40].

While the estimates of acceptability in the available studies is relatively high, the quantity of included texts underlines the dearth of knowledge we have about chlamydia, gonorrhea, syphilis, and trichomoniasis vaccines. As much of this research is at least one, if not two decades old, there could be changes in vaccine acceptability not reflected in the available data. Of note, all the included studies had data collection prior to the COVID-19 pandemic, and the pandemic could have impacted patterns of adult vaccine hesitancy (e.g., as seen for pediatric vaccinations [42]).

There are some limitations to the generalizability of these studies. The studies are in the United States [34–37, 40, 41] and in Canada [38, 39]. Previous cross-national surveys have found substantial differences across countries for influenza [43] and COVID-19 [44] vaccine acceptance. Previous systematic reviews of HPV [45], COVID-19 [46, 47], and influenza [48] vaccines show that acceptability and uptake of vaccines could vary based on factors that substantially differ across countries, including insurance / health care systems, religion, trust in authorities, political polarization in vaccination, and attitudes towards sexual behaviors.

Studies of the HPV vaccine [32, 45], the mpox vaccine [49], and other vaccines for MSMs like hepatitis A and hepatitis B [50] might be the closest analogues for understanding the future roll-out of another STI vaccine. Notably, consistent and strong recommendations from health care providers are one of the most important factors in deciding whether someone will accept a vaccine [45, 50]. Convenience of access to the vaccine site is important [32]. For the mpox vaccine, greater perceived susceptibility, more cues for action, and more convenient access did increase vaccine uptake [49]. However, the proportion who rejected a vaccine offered on site at a clinic visit can still be relatively high – 15% in one study of mpox [49]. Mathematical modeling could determine whether that percentage could have a substantial epidemiological consequence.

Systematic reviews can be limited in their ability to explain the reasons behind significant findings [51] due to lack of consistent measurement of various issues across studies. We acknowledge in this systematic study the lack of consistent measurement of certain factors that likely had a large impact on individual and collective levels of decision-making. Stigma in particular is important to define and research as it affects testing and treatment of STIs [52–54], and could affect vaccine distribution [55]. Parents also could be worried that STI vaccination could affect their child’s sexual behaviors [31], although research does not suggest this occurs [56].

There is also the concern that the acceptability measures we do have are skewed towards those who are more willing to seek medical care and who have better access to medical care. Many of the studies sampled participants from healthcare settings [36, 39–41], and the rest sampled from larger health-based studies [34, 35, 37, 38]. As such, there is the possibility that these studies are subject to selection bias, and might not accurately represent communities with lower access to healthcare or higher distrust in the healthcare system, including racial and ethnic minorities, sexual and gender minorities, low-income individuals, and non-English speakers [57–60]. As such, additional research into vaccine acceptability among these populations, especially sampled outside of healthcare settings, could be crucial to having a better understanding of vaccine acceptability.

There is also a need to better understand how vaccine characteristics might impact receptivity [23]. As shown with HPV vaccines, acceptability does not necessarily equate to uptake, initiation is often higher than completion, and timeliness of completion is not always to schedule [50, 61, 62]. The included studies inquired about whether or not participants would receive certain STI vaccines. Differences between acceptance and uptake could result from the following reasons: yet-unknown features of the vaccination program, like number of doses required [63]; cues to action and vaccination planning [64]; social desirability bias in responding to questions in a certain way; and other factors. Further research into these and additional vaccine characteristics’ effects on vaccine acceptability is necessary to accurately predict vaccine uptake.

### Limitations

One notable limitation of this study is that inclusion was limited to publications written in English, which could have excluded publications of interest written in other languages. Another limitation is breadth of search. While scoping analyses are meant to be comprehensive, they are not always exhaustive, and there is the potential that the databases we utilized were not fully representative of the literature relevant to our study. In the interest of the resource and time limits on this examination, we limited our Google Scholar search results to title searches only, cutting down on the number of texts we collected, but also potentially missing some texts of interest.

## Conclusions

Just three years after Abraham et al. published the results of the Phase I trial of their Chlamydia vaccine candidate [11] and with the potential of more STI vaccine candidates on the horizon [24], evidence for STI acceptability is now more important than ever. While the texts included in this scoping analysis demonstrate high vaccine acceptability in their study populations, more research is needed to achieve a robust understanding of the public’s willingness to receive gonorrhea, chlamydia, syphilis, and trichomoniasis vaccines. More research outside of the United States and Canada, additional research into populations with reduced healthcare access, and investigation into the effects of vaccine characteristics on acceptability are needed before we can adequately prepare for future STI vaccine rollouts.

### Electronic supplementary material

Below is the link to the electronic supplementary material.

Supplementary Material 1

Supplementary Material 2

## Data Availability

All data generated or analysed during this study are included in this published article.

## Abbreviations

STI: Sexually Transmitted Infection
PID: Pelvic Inflammatory Disease
AMR: Antimicrobial resistance
HPV: Human Papillomavirus
MSM: Men who have Sex with Men
HIV: Human Immunodeficiency Virus
CAS: Condomless Anal Sex
PrEP: Preexposure Prophylaxis
