# Supplementary material for: Public willingness to receive chlamydia, gonorrhea, syphilis, and trichomoniasis vaccines: a scoping review
Source: BMC Health Serv Res. 2023 Nov 23;23:1290. doi: 10.1186/s12913-023-10334-9 (PMC10668406; doi:10.1186/s12913-023-10334-9)
Supplement: Supplementary file 1 — Supplementary Material 1 [file 12913_2023_10334_MOESM1_ESM.docx]

**Additional File 1.** The search strategy for the scoping review.

(TITLE-ABS-KEY("Syphilis" OR "Treponema pallidum" OR "Treponemal Infection*" OR “Infection, Treponemal” OR “Infections, Treponemal” OR "Gonorrhea" OR "Neisseriaceae Infection*" OR "Neisseriaceae Infection*" OR "Neisseria gonorrhoeae" OR “Infection, Neisseriaceae” OR “Infections, Neisseriaceae” OR "Chlamydia" OR "Chlamydia trachomatis" OR "Trichomonas Infection*" OR "Trichomonas Infection*" OR “Trichomoniasis” OR "Trichomonas vaginalis" OR “Sexually Transmitted Disease, Bacterial” OR “Sexually Transmitted Diseases, Bacterial” OR “Bacterial Venereal Disease*” OR “Disease, Bacterial Venereal” OR “Venereal Disease, Bacterial” OR “Venereal Diseases, Bacterial” OR "Bacterial STI" OR "Bacterial Sexually Transmitted Infection*" OR "Curable STI" OR "Curable Sexually Transmitted Infection*" OR "Curable STD*" OR "Curable Sexually Transmitted Disease*" OR "Bacterial STD*" OR "Bacterial Sexually Transmitted Disease*")) AND (TITLE-ABS-KEY("vaccination hesitanc*" OR "vaccination hesitanc*" OR "vaccination refusal*" OR "vaccination refusal*" OR “Hesitancy, Vaccination” OR “Vaccine Hesitanc*” OR “Hesitancy, Vaccin*” OR “Vaccine Delay*” OR “Delay, Vaccine” OR “Delays, Vaccine” OR “Vaccination Delay*” OR “Delay, Vaccination” OR “Delays, Vaccination” OR “Refusal, Vaccination” OR “Refusals, Vaccination” OR “Vaccine Refusal*” OR “Refusal, Vaccine” OR “Refusals, Vaccine” OR (vaccin* AND attitude*) OR (vaccin* AND interest*) OR (vaccin* AND concern*) OR (vaccin* AND reluctan*) OR (vaccin* AND feel*) OR (vaccin* AND knowledge) OR (vaccin* AND Opinion) OR (vaccin* AND Willing*) OR (vaccin* AND Willing*) OR (vaccin* AND recepti*) OR (vaccin* AND Hesitan*) OR (vaccin* AND thoughts) OR (vaccin* AND sentiment*) OR (vaccin* AND stigma) OR (Immunization* AND attitude*) OR (Immunization* AND interest*) OR (Immunization* AND concern*) OR (Immunization* AND reluctan*) OR (Immunization* AND feel*) OR (Immunization* AND knowledge) OR (Immunization* AND Opinion) OR (Immunization* AND Willing*) OR (Immunization* AND Willing*) OR (Immunization* AND recepti*) OR (Immunization* AND Hesitan*) OR (Immunization* AND thoughts) OR (Immunization* AND sentiment*) OR (Immunization* AND stigma) OR (“Active Immunization*” AND attitude*) OR (“Active Immunization*” AND interest*) OR (“Active Immunization*” AND concern*) OR (“Active Immunization*” AND reluctan*) OR (“Active Immunization*” AND feel*) OR (“Active Immunization*” AND knowledge) OR (“Active Immunization*” AND Opinion) OR (“Active Immunization*” AND Willing*) OR (“Active Immunization*” AND Willing*) OR (“Active Immunization*” AND recepti*) OR (“Active Immunization*” AND Hesitan*) OR (“Active Immunization*” AND thoughts) OR (“Active Immunization*” AND sentiment*) OR (“Active Immunization*” AND stigma) OR (Inoculation* AND attitude*) OR (Inoculation* AND interest*) OR (Inoculation* AND concern*) OR (Inoculation* AND reluctan*) OR (Inoculation* AND feel*) OR (Inoculation* AND knowledge) OR (Inoculation* AND Opinion) OR (Inoculation* AND Willing*) OR (Inoculation* AND Willing*) OR (Inoculation* AND recepti*) OR (Inoculation* AND Hesitan*) OR (Inoculation* AND thoughts) OR (Inoculation* AND sentiment*) OR (Inoculation* AND stigma)))
