# Supplementary material for: Public willingness to receive chlamydia, gonorrhea, syphilis, and trichomoniasis vaccines: a scoping review
Source: BMC Health Serv Res. 2023 Nov 23;23:1290. doi: 10.1186/s12913-023-10334-9 (PMC10668406; doi:10.1186/s12913-023-10334-9)
Supplement: Supplementary file 2 — Supplementary Material 2 [file 12913_2023_10334_MOESM2_ESM.docx]

**Additional File 2.** Quality Assessment evaluations for the included texts. The criteria include questions 1, 2, 7, and 8 from JBI’s *Checklist for Analytical Cross Sectional Studies* critical appraisal tool [1] with clarifying criteria utilized by the reviewers.

|  | Were the criteria for inclusion clearly defined?  (Clarifying criteria: Mentions inclusion and exclusion criteria) | Were the study subjects and setting described in detail? (Clarifying criteria: Mentions when and where the study took place, as well as the sampling frame) | Were the outcomes measured in a valid and reliable way? (Clarifying Criteria: Mentions how they measured acceptability and where they got the question from/how it was developed) | Was appropriate statistical analysis used? (Clarifying Criteria: Provides measures of precision or dispersion of vaccine acceptance) |
| --- | --- | --- | --- | --- |
| Abara 2022 | x | x |  |  |
| Trent 2016 | x |  |  |  |
| Zimet 2002 | x |  |  |  |
| Bonney 2007 | x | x | x |  |
| deWaal 2022 | x | x | x | x |
| Plotnikoff 2020 | x | x | x |  |
| Mays 2004 | x | x |  |  |
| Zimet 2005 | x |  | x |  |

[1. Moola S, Munn Z, Tufanaru C, et al. Chapter 7: Systematic reviews of etiology and risk. In: Aromataris E, Munn Z, eds. *Joanna Briggs Institute Reviewer’s Manual*. The Joanna Briggs Institute; 2017. https://reviewersmanual.joannabriggs.org/](https://www.zotero.org/google-docs/?8wxkut)
